# Industrial Multiple-Effect Fractional Condensation under Vacuum for the Recovery of Hop Terpene Fractions in Water

**DOI:** 10.3390/foods12081716

**Published:** 2023-04-20

**Authors:** Lorenzo Lamberti, Luisa Boffa, Giorgio Grillo, Stefano Concari, Francesca Cavani, Giancarlo Cravotto

**Affiliations:** 1 Dipartimento di Scienza e Tecnologia del Farmaco, University of Turin, via P. Giuria 9, 10125 Turin, Italy; lorenzo.lamberti@unito.it (L.L.); luisa.boffa@unito.it (L.B.); giorgio.grillo@unito.it (G.G.); 2 Baladin S.S. Agricola, via Carrù 23, 12060 Piozzo, Italy; 3 Tropical Food Machinery, via Stradivari 17, 43011 Busseto, Italy; stefano.concari@tropicalfood.net (S.C.); francesca.cavani@tropicalfood.net (F.C.)

**Keywords:** green extraction, hops (*Humulus lupulus* L.), multiple-effect fractional condensation under vacuum, terpenes, industrial-scale extraction, HS-SPME/GC-MS, dry hopping

## Abstract

The inflorescences of *Humulus lupulus* L. are the most valuable ingredient in the brewing industry. Only female cones are used as their bitterness and aroma, much associated with beer, are granted by the production of resins and essential oils, respectively. The traditional brewing process for the extraction of the organic volatiles in hops is called dry hopping. It consists of extended maceration at low temperature after the fermentation phase. New extraction technologies can improve extraction rates and product quality while saving time and money. This article proves that multiple-effect fractional condensation under a vacuum is suitable for flavouring applications and especially for performing dry hopping without contamination risks and reductions in hop amounts. This technique leads to the recovery of aqueous aromatic fractions that are very rich in hop sesquiterpenes and monoterpenes. These suspensions are extremely stable when stored at 5–8 °C and avoid degradation even after several months. This feature is crucial for the marketing of non-alcoholic beverages, where the dilution of essential oils is otherwise problematic.

## 1. Introduction

Every year, more than 130,000 tonnes of hop cones are processed into hop pellets to supply the growing brewing industry [1]. Hop cones are the inflorescences of the female plant *Humulus lupulus* L., which belongs to the *Cannabaceae* family. This plant is a perennial climbing vine, native to Europe, Southwest Asia, and North America, and requires a temperate climate [2]. There is only one harvest per year, and this occurs between the end of August and the first weeks of September. Almost 97% of hops produced are destined for brewing purposes [3]. The USA leads world production with 44.3 tonnes, followed by Germany and the Czech Republic with 39.0 tonnes and 6.1 tonnes, respectively [4]. The glands of the female plant produce and secrete lupulin, a small yellowish resinous powder found at the base of the inflorescence brackets. These glands have a high concentration of essential oils and resins, which are responsible for the organoleptic properties of beer [5]. They serve as storage for the resins and essential oils (EO) synthesised by the plant [6]. The production of these compounds depends on the hop variety and plays a crucial role in the final fragrance [7]. At the end of the harvest, the hop cones have a water content of over 75%. In this state, decomposition, and mould growth can occur very quickly, so it is important to dry the material as soon as possible. To preserve the EO and avoid losses, it is important to keep the temperature as low as possible throughout the process. When moisture content reaches 10%, the matrix can be stored for one year in a controlled atmosphere (ca. 6 °C) to prevent oxidation. Normally, however, the cones are converted into pellets, as they have a much higher density and are more practical to use. Pelletisation is carried out in two successive steps: first, the cones are crushed and then conveyed to an extruder, which produces the typical cylindrical shape of 2–3 cm long and 5 mm in diameter [8]. 

Hop cones are of complex chemical composition, as listed in Table 1 [2,9,10]. Since the cones are mainly composed of bracts, the amounts of proteins, cellulose, and polyphenols is very high compared to those of resins and EO, which are the active aroma molecules. This fact shows how strong these molecules are in terms of flavour, especially in beer, where the hops represent less than 5% of the ingredients used but are still able to characterise the final product to such an extent.

The most critical constituents for brewers are resins and EO. The first class of compounds can be divided into hard and soft resins, depending on their solubility in hexane. The latter are divided into α- and β-acids. The α-acids are primarily responsible for the bitter taste in beer [11].

The EO are, by definition, the lipophilic volatile fraction of the hops and are responsible for the aroma imparted to the beverage. The aroma can change significantly depending on plant variety and growing environment [12]. Many studies have reported that this volatile fraction is a complex mixture of more than 200 components [13], and even more recently, the use of comprehensive multidimensional gas chromatography and a flame ionisation detector (FID) has led to the detection of about 1000 different compounds [14]. Due to the low concentrations involved, the human gustatory system cannot perceive most of these molecules. The chemical composition of the EO of *Humulus lupulus* L. can be divided into three main groups: hydrocarbons, oxygenated compounds, and sulphur-containing components [15,16]. The hydrocarbons group is the most significant and accounts for about 60–75% of EO and can be further divided into monoterpenes and sesquiterpenes, depending on the number of isoprene units (two or three, respectively). The monoterpenes are less numerous, although they include β-myrcene, which is generally the most abundant EO, accounting for 30–60% of the total [17]. Another detectable monoterpene is limonene, which is only present in some hop varieties, but provides a very pungent characteristic flavour [13]. In fact, the Citra hop variety, bred by Yakima Chief Hops, was given its name because of the citrus aromas that develop during ripening. The second class of compounds that make up the EO are sesquiterpenes, which are characterised by a higher boiling point; the main components are humulene, caryophyllene, and farnesene. Due to their chemical nature, the hydrocarbon group is more volatile than other EOs with the same molecular weight and has higher oxidation and polymerisation reaction rates [18].

The oxygen-bearing components can make up to 30% of the volatiles fraction [8], and their composition is far more complex, including alcohols, aldehydes, acids, ketones, epoxides, and esters [2,19]. This category can be further divided into volatiles and non-volatiles depending on boiling point: generally, if the component has a higher boiling point than humulene, it is considered non-volatile. Even if this group is present in lower concentrations than the hydrocarbons, it is significant for the final aroma of the hops [20]. In this category, we find compounds, such as linalool and geraniol, which bring a strong floral scent that characterises many hops, such as the Cascade and the Continental variety [21,22]. Sulphur-containing EO are present as traces in the volatiles, and sulphur flavours are generally considered defective in beer. However, those aromas are generally caused by the stressed fermentation of *Saccharomyces cerevisiae* or a low-quality malt [16]. Unfortunately, these sulphur-containing components have a low odour threshold and can influence the final hop aroma [23,24,25]. EO can deteriorate over time, causing changes in the flavouring fingerprint that are mainly due to oxidation reactions. For this reason, it is always suggested that hops are maintained in an oxygen-free atmosphere at low temperatures, even during the processing from hop cones to pellets. It is worth noticing that hops are not only a flavouring agent but that their components are investigated for a wide range of applications, such as therapeutic, cosmetic, and nutritional uses [26,27]. They also show interesting bioactivity, such as antioxidant, antimicrobial, antiviral [28,29], antitumoral [30,31], and pest-control features [32].

The conventional extraction of EO from the hops consists of steam distillation that lasts up to 5 h [33]. This process is very time and energy-consuming and uses a traditional heating method. In the last decade, companies have been investing considerable resources into the optimisation of their effective practices thanks to an increasing concern about the environmental situation [34]. These new strategies, which follow the “green extraction” approach [35], must enhance process intensification and extraction efficiency while using safe and sustainable solvents. One suitable example is MW-assisted hydrodistillation, which has already been tested on hops and *Cannabis sativa* L. in our research group [36,37]. The most commonly used technique for the industrial extraction of the volatile fraction on *Humulus lupulus* L. is supercritical CO_2_ (sc-CO_2_). This technology exploits the polarity of the supercritical fluid, which, in this state, is capable of extracting both the resins and the volatile fraction [38,39,40]. This method gives good yields, and the product is stable and can be easily introduced during the brewing process since the solvent is a component of the beer itself. The main drawback lies in the fact that the extract is a complex mix of many different compounds and also includes resins with high bittering power.

Based on the principle of adsorption/absorption and desorption, headspace solid-phase microextraction (HS-SPME) uses a coated fibre to trap and concentrate volatile and semi-volatile analytes from the vapor phase above a sample in a static or dynamic headspace process [41]. HS-SPME is a simple aroma extraction technique that extracts a wide boiling-point range of volatile compounds without artifact formation and integrates sample preparation, extraction, concentration, and the introduction of the sample into gas-chromatography (GC). This technique, generally applied to samples with concentrations in the ppb–low ppm range, has been validated for numerous applications and, in particular, for reliable quantitative analysis since a direct proportion between the amount of analyte extracted by the fibre and its initial concentration in the sample matrix has been demonstrated [42]. In order to overcome problems with extraction reproducibility and therefore optimise analyte recovery, the polymeric fibre coating and coating volume, sample preparation, extraction time (sample/HS equilibrium), equilibration time (HS/fiber equilibrium), and desorption time must be optimised. Several studies have investigated the aroma profile of hops using SPME extraction, in which the semi-quantitative analysis of compounds is performed using the total peak area [43], relative peak area (%) [44,45,46], and peak-area ratios of analyte internal standards (IS) [47,48]. In 2021, Su and Yin quantitated hop aroma compounds using a combination of stable isotope dilution analysis (SIDA) and standard addition method (SAM) in order to eliminate the matrix effect [49]. 

The present study tests Multiple-Effect Fractional Condensation Under Vacuum (MEFCUV) in recovering the lipophilic volatile fraction from hops. This technique is not only able to selectively extract the terpenes but also to separate the volatiles into two fractions, one enriched in sesquiterpenes, such as caryophyllene, farnesene, and humulene, and the other enriched in monoterpenes. The production of separated extracts increases potential applications in brewing as well as in other beverages and perfumes. Myrcene, for example, is often used in the perfume industry, especially in men’s fragrances, because of its pungent aroma. In this case, when the floral and grassy scents of sesquiterpenes may be undesirable, a fragrance that is heavily enriched with myrcene would be preferable. HS-SPME has been applied to distilled hop fractions to determine their qualitative and semi-quantitative composition in volatiles. For this purpose, gas chromatography was coupled with mass spectrometry (GC-MS), and an IS was added to the samples before SPME extraction [47].

## 2. Materials and Methods

### 2.1. Vegetal Matrix

The hops used belong to the Citra and Chinook varieties. The Chinook hops were harvested at the end of August 2020 in the Piedmont region (Italy). After harvesting, the cones were dried for 12 h at 42 °C, which decreased the average humidity to 8%. The material was left for 24 h at room temperature before pelletisation. Dried cones were left to rest for 24 h, and their humidity rose to 10/11%, and the hops then underwent pelletisation. This process started with a fine mincing of the cones, and the collected powder was then pressed through an extruder to create the pellets. The temperature increased substantially during pelletisation and reached 50 °C. At the end of processing, the hops were stored under a nitrogen atmosphere at 6 °C for their preservation. Citra hops were bought from the Yakima Chief Hops company and kept at 6 °C during storage. The hops present themselves in pellet form, a small cylinder of 1/2 cm in length and 0.5 cm in diameter. The water content was evaluated via thermogravimetric analysis by leaving an average weight of 1.5 g of biomass at 100 °C overnight and measuring the relative weight loss. The tests were performed in triplicate, and the results are shown in Table 2. The humidity level of the Citra variety is in line with the technical sheet of Yakima Chief Hops for this product.

### 2.2. Chemical Standards and Reagents

Toluene (ACS grade, ≥99%) and the analytical standards (myrcene, caryophyllene, farnesene, humulene) used for GC-MS analyses were purchased from Sigma-Aldrich (St. Louis, MO, USA).

### 2.3. Aroma Recovery Unit

**Figure 1:**
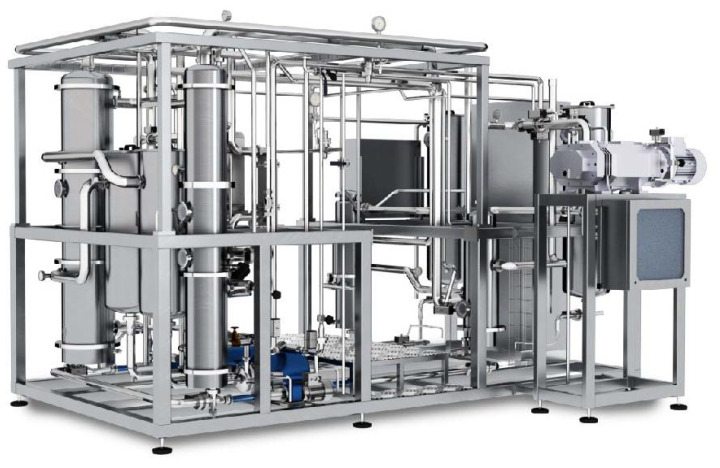
Multiple-Effect Fractional Condensation Under Vacuum (MEFCUV) (Tropical Food Machinery, Busseto Italy).

The extraction was carried out using Multiple-Effect Fractional Condensation Under Vacuum (MEFCUV) (Figure 1), produced by the company Tropical Food Machinery (Busseto, Italy).

**Figure 2:**
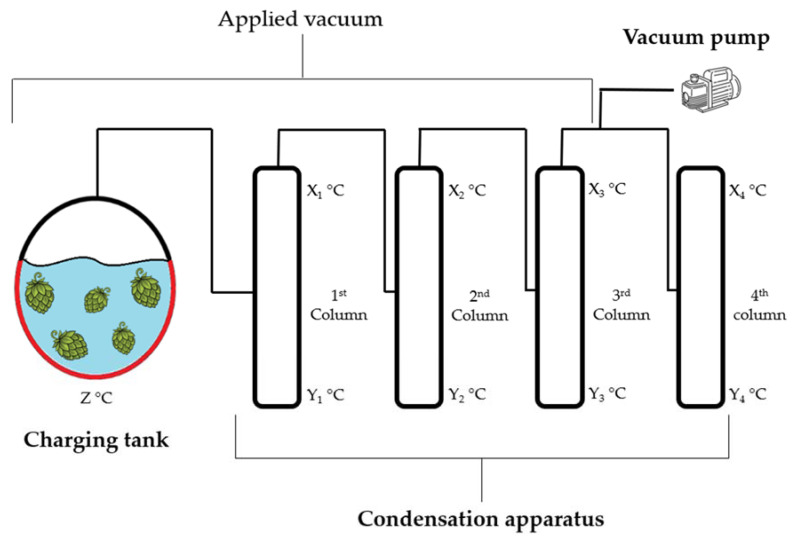
Scheme of the MEFCUV system. Starting from the left: charging tank; condensation apparatus; vacuum pump. Temperature in each part of the system can be regulated independently.

The extractor is made up of two major components: the charging tank and the condensation apparatus. The charging tank has an internal volume of approximately 150 L and a mechanical paddle mixer with scrapers. The main tank has an outside jacket connected to a vapor generator to heat the vessel. The vessel has the purpose of creating a vapor that is rich in flavour compounds and is able to work under a vacuum, thus reducing the boiling temperature. The charging tank is connected to the condensation apparatus, which is composed of four condensation columns refrigerated at the top and heated at the bottom to enhance fractional distillation. The whole system is connected to a vacuum pump that can reach −900 mbar, which is itself connected to the charging tank and to the first three columns. A simplified scheme of MEFCUV is reported in Figure 2.

### 2.4. Hops Processing

For the first batch, 12 kg of Chinook pellet hops were loaded into the charging tank, and 12 L of water was added to rehydrate the biomass (1:1 S/L ratio). Soaking was accelerated by homogenisation for 20 min. When all of the hops were suspended, the chamber was heated to 50 °C. The condensation columns were set at different temperatures, as shown in Table 3. After 50 min of extraction, the temperature inside the columns started to increase due to chiller-dimensioning issues. The extraction was stopped to contain the leakage of volatiles from the last column. The water from the condensation apparatus was recovered and stored at 6 °C. The extracts collected from the second column were labelled as HCH (Heavy volatiles Chinook), whilst LCH (Light volatiles Chinook) represent the merged product of the third and fourth columns. The sample recovered from the first condensation step was discarded due to excessive dilution.

The second extraction was performed at a higher average temperature in each condensation column, as shown in Table 4, to reduce the demand on the chiller. A total of 10 kg of Citra hops was loaded with 10 L of water for rehydration and were left to soak for 20 min under agitation. The extraction was carried out for 90 min. The water from the condensation apparatus was recovered and stored at 6 °C. The extracts collected from the second column were labelled as HCT (Heavy volatiles Citra), whilst LCT (Light volatiles Citra) represent the merged product from the third and fourth columns. The sample recovered from the first condensation step was discarded due to excessive dilution.

### 2.5. HS-SPME/GC-MS of Hop Samples

The condensed water collected from each sample was placed inside a separation funnel for 2 h at room temperature. This procedure was performed to ensure that the suspension volatiles inside the water were stable and did not create a second lipophilic phase at the top. The SPME holder for manual sampling and the Divinylbenzene/Carboxen/Polydimethylsiloxane SPME fibre (1-cm 50/30, DVB/CAR/PDMS) used were purchased from Supelco (Bellefonte, PA, USA). Clear crimp top glass vials (20.0 mL) and silver aluminium caps with PTFE/silicone septa were obtained from Agilent Technologies (Palo Alto, CA, USA). The fibre was first conditioned in the GC injection port at 250 °C to remove fibre contaminants and cleaned prior to each extraction in the hot injection port for 15 min. Analyses were performed in triplicate and expressed as means ± standard deviation (S.D.).

#### 2.5.1. HS-SPME Parameters

An aqueous toluene solution was used as an internal standard (IS). It was prepared at 86.7 mg/L for the HS-SPME analysis. Distilled fractions (500 μL) (HCH, LCH, HCT, LCT) were placed into a headspace 20-mL vial followed by the addition of 3.00 mL of toluene IS solution at 86.7 mg/L in water, while 3 mL of a 1:1 diluted toluene IS solution (43.4 mg/L) was added to the ground pellets (10 mg). The vials were clamped and equilibrated for 10 min in a hot water bath at 50 °C. After equilibration, a preconditioned 1-cm 50/30 DVB/CAR/PDMS fibre was exposed to the headspace of the clamped vial for 30 min at 50 °C [49]. The accumulated analytes were recovered via thermal desorption directly into the GC injector port for 10 min at 250 °C.

#### 2.5.2. GC-MS Parameters

The GC-MS analyses were performed in an Agilent Technologies 6850 Network GC System, using a 5973 Network Mass Selective Detector, a 7683B Automatic Sampler (Santa Clara, CA, USA), and a Mega 5-MS capillary column (5% Phenyl, 95% Methyl Polysiloxane, 30 m × 0.25 mm i.d., 0.25 μm film thickness) (Mega S.r.l., Legnano, Italy).

The injector was kept at 250 °C in split mode with a 10:1 split ratio. The oven temperature was programmed to start from 40 °C, was held for 2 min, then moved to 200 °C at 4 °C/min, and then from 200 °C to 260 °C at 20 °C/min and held for 5 min. Helium was used as the carrier gas at a constant flow rate of 1.2 mL/min (average velocity 40 cm/s).

Conditions for the MSD were as follows: detector temperature at 280 °C, MS source at 230 °C, MS quadrupole at 150 °C, mass range, 15–400 amu.

#### 2.5.3. Identification and Quantification

Compounds were considered to be positively identified when electron-impact (EI) mass spectra matched with Wiley7n and NIST11 libraries with a minimum quality of 90%. The compounds that failed to meet the above criteria were considered to be tentatively identified. The identification of myrcene, caryophyllene, farnesene, and humulene was performed based on standard retention times. 

The semi-quantitative analysis of flavour compounds (FC) in hop samples was performed based on toluene amount (mg), used as an IS [47], using the following formula:

µg _IS_ × Area _FC_/Area _IS_.

## 3. Results

### 3.1. Aroma Recovery Unit

The volume of the condensed water for each column at the end of extraction is reported in Table 5.

As can be seen, there is no proportion between the volume collected in each column between the Chinook and Citra extraction. During the first process, the temperature inside the columns increased significantly, causing a net loss of condensed water. From Table 5, it is clear that the loss of temperature control led to higher recovery in the last columns due to lower condensation in the first and second columns, which were unable to efficiently condense the vapor stream.

The first organoleptic impressions of the condensed extracts indicated that the first column in the Citra and Chinook samples had no flavour. On the other hand, the condensed extract from the second, third, and fourth columns had a pleasant aroma, which was much stronger for Citra. This difference can be explained by the issue encountered by the recovery unit during the first extraction, which led to an overall aroma loss. During extraction, the hops lost almost all the water that was added at the beginning of the process. Vacuum evaporation at low temperatures resulted in the deposition of hops on the internal surfaces, enhancing the extraction rate by increasing the exchange area. The final moisture content of the hops was 16.9 ± 0.2 (*w*/*w*%), and no burnt areas were observed. The aroma of the residual hops was very faint and grassy, without peculiar characteristics. In the supporting material (Table S1), an overview of the typical compounds that characterise the hops matrix is displayed. The water collected during the Chinook extraction was utterly transparent in the first two columns, while light yellow turbidity was found on the samples collected from columns 3 and 4. The samples collected from the Citra extraction showed much higher turbidity and yellow colour with some opalescent reflections on the surface. The flavours were much more potent, and the extract collected from the third and fourth columns were indistinguishable, although more piney and pungent compared to the second column.

As mentioned above, the temperature increased in the last column during Chinook extraction, leading to a significant loss in volatile compounds. This phenomenon was visible as a thick hoppy-smelling vapor cloud started to exit from the head of the last section of the condensation apparatus. During the second extraction, the protocol was modified, with columns temperatures that were sustainable for the chiller being set. Hence, the energy demands on the chiller were reduced, and the desired results were achieved. In fact, only a very light scent of the hop was perceivable during the process. At the end of the extraction process, the hops maintained their vivid green colour and had a low concentration of humidity, although no burnt biomass was found. The spent hops lost their characteristic flavour, which changed to a slight scent of grass.) This empiric evaluation suggests that aroma extraction was successful, and few volatiles remained in the biomass. Further tests can be performed to optimise the extraction and thus reduce the overall treatment time.

### 3.2. HS-SPME/GC-MS Analyses

According to several articles, the DVB/CAR/PDMS fibre was chosen for hop-sample analyses [43,48,49]. Toluene was used as an IS for the semi-quantitative analyses because of its good solubility in water (526 mg/L at 25 °C, Pub Chem) and based on several experiments performed by the authors in the semi-quantitation of the profiles of fruit aromatic fractions extracted with the same equipment. Procedure parameters, such as the sample amount (mg or mL), IS concentration (mg/L), and amount (mL) added to the samples, were optimised to achieve the best reproducibility in results.

**Figure 3:**
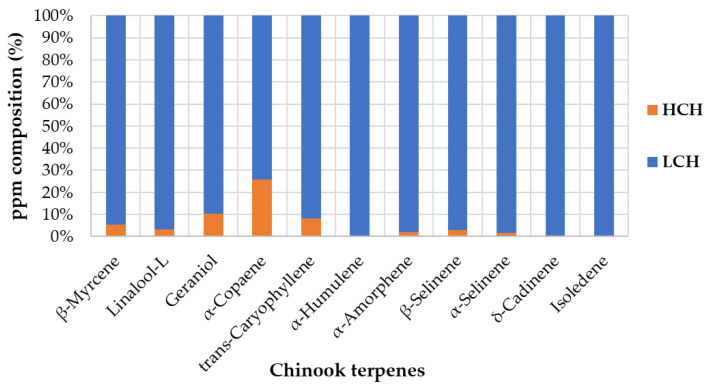
Hundred percent stacked column representation of the average ppm data from Table 6.

The data recovered from the triplicate analysis are available in the supporting materials (Tables S2–S4). Table 6 reports the quantitative results for the HCH and LCH samples with the S.D. of each compound. For the sake of comparability, the study highlights the key molecules responsible for the peculiar flavour found in both extracts. Figure 3 shows a graphical representation of the abundance of each compound in the HCH and LCH samples and helps to underline the significant disproportion between the abundances of recovered compounds.

**Figure 4:**
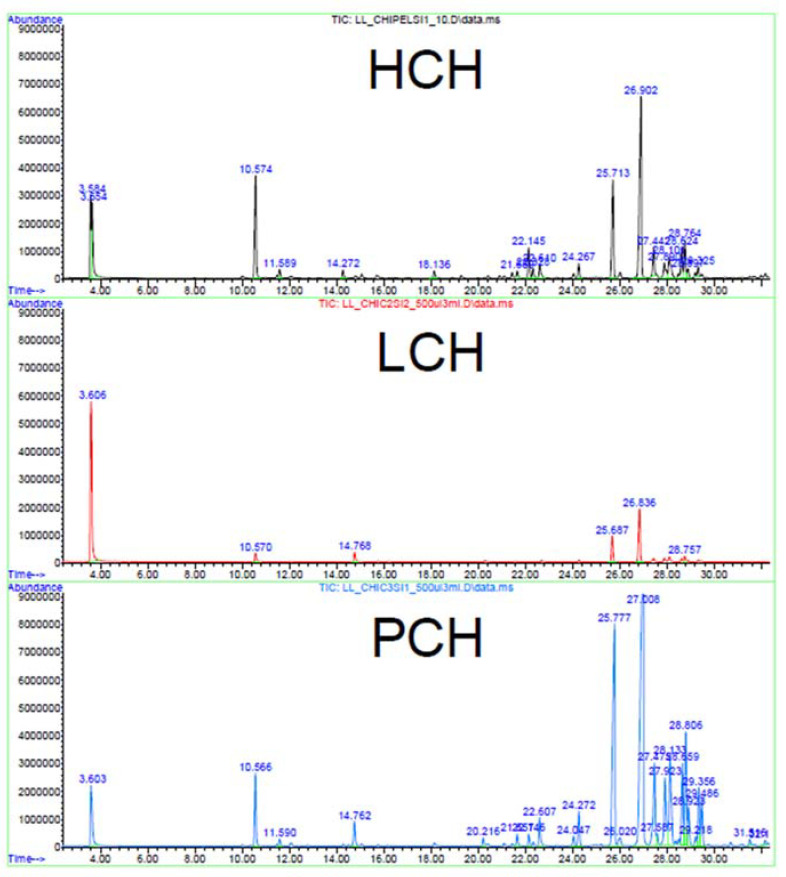
Superimposition of HS-SPME/GC-MS chromatograms of the HCH, LCH, and PCH samples with the respective retention time for each compound.

The quantity of the volatiles condensed in the HCH fraction is poor due to the reduced extraction time and the chiller issues faced during the procedure. The heavy volatiles that condensed in column 2 shifted again to the vapor state when the temperature increased, thus moving to the subsequent columns. The lower concentration of compounds in the HCH fraction caused a decrease in the matching quality of the less abundant peaks by MS libraries (lower than 90%) compared to LCH and both HCT and LCT. In addition, the triplicates did not show a complete overlapping of peaks that were present only in traces, although, as can be appreciated in Figure 4, there is a clear overlap of the major peaks in all three samples. This detail, alongside the comparison with the compounds recovered from the Chinook pellet (PCH), proves that the extracts maintained the volatile compounds present in the starting biomass.

Table 7 reports the semi-quantitative results for the HCT and LCT samples with the standard deviation of each compound. As for the Chinook samples, the key molecules responsible for the peculiar flavour found in both extracts have been highlighted for the sake of comparison. All of the data collected can be viewed in the supporting material (Tables S5 and S6).

**Figure 5:**
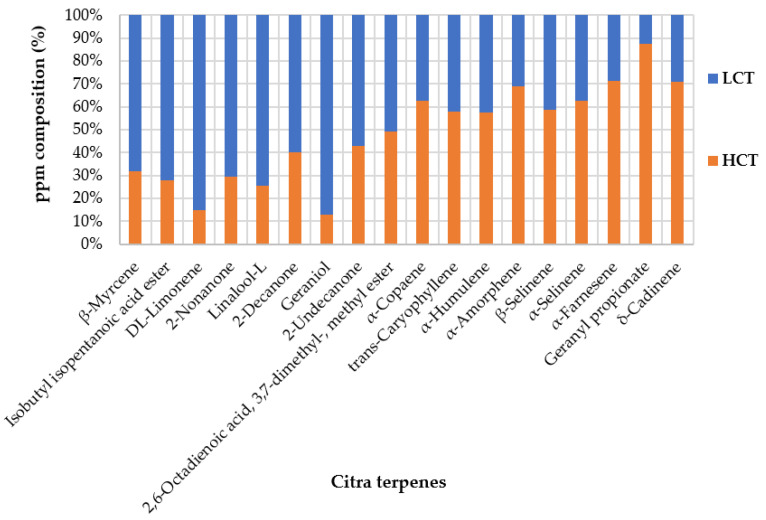
Hundred percent stacked column representation of the average ppm data from Table 7.

**Figure 6:**
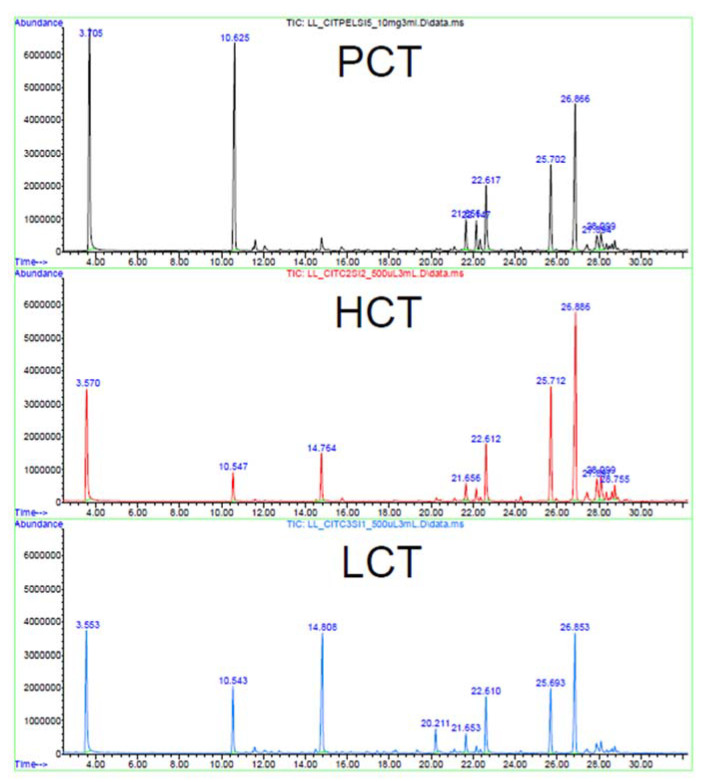
Superimposition of HS-SPME/GC-MS chromatograms of the HCT, LCT, and PCT samples with the respective retention time for each compound.

Figure 5 reports the average percentage abundance of each compound found in the HCT and LCT samples. The lighter monoterpene compounds, placed on the left side of the graph, have higher concentrations in the LCT samples. Meanwhile, the sesquiterpene fraction that starts from α-Copaene has higher concentrations in the HCT samples. Figure 6 shows a superimposition of the chromatograms of the Citra Pellet (PCT), HCT, and LCT samples. As discussed in Figure 4, the extracts recovered have a high correspondence with the peaks of the pellet biomass.

The separation of the two fractions increases the possible applications of the extracts. It would be possible to increase the separation of the monoterpenes and sesquiterpenes by acting on the temperature set on each column. A higher temperature in column 2, for example, would shift the condensation equilibrium to the vapor phase, especially in the case of the monoterpenes, allowing them to pass forward to columns 3–4. This modification would cause an increase in the temperature and vapor stream that arrives at the last columns, thus causing the chiller to undergo higher strain to maintain the parameters. Unfortunately, due to the limitations in chiller power, it was not possible to emphasise this separation, as observed in the first extraction with the Chinook pellet hops. The separated enrichment of the monoterpene and sesquiterpene fractions creates two different fragrances that could be more suitable than a single solution with both. For example, the perfume industry has a high myrcene demand since it is used for its characteristic hearty and pungent piney flavour, widely used in men’s fragrances. In this case, the LCT fraction can be exploited alone due to its lower percentage of sesquiterpenes, such as caryophyllene humulene and farnesene, which are more floral and fruity. Further tests could be performed to optimise the extraction time and optimal temperature setting for each column if a higher separation of monoterpenes and sesquiterpenes is requested. 

**Figure 7:**
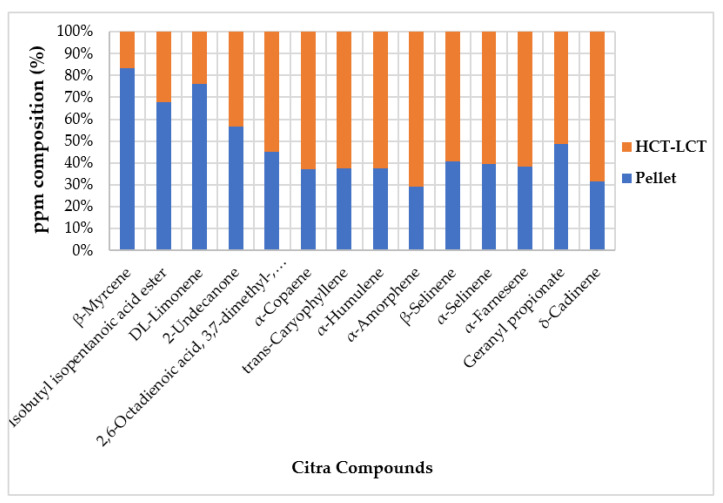
Comparison of the percentage area peaks of the data recovered from the Citra pellets and the weighted average of the HCT and LCT samples.

In order to evaluate the quality of the recovered extracts, the merged HCT and LCT samples were compared with PCT by means of an HS-SPME/GC-MS analysis. All of the data recovered from the triplicate analysis are shown in the supporting materials (Tables S5–S7). To evaluate whether the same ratios between the compounds were maintained in the final product, we used the percentage area peaks of the pellets and a weighted average of the HCT and LCT area peaks (Figure 7).

From this histogram, it is clear that there is a high disproportion between the compared data. As presumed, there is a higher concentration of monoterpenes in the pellet. This result is not surprising since the monoterpenes exhibit a lower boiling point and thus are the hardest to condense and the easiest to lose. During extraction, the slightly pungent flavour that came out of the head of the fourth column can be associated with the lighter compounds (β-myrcene, isobutyl isopentanoic acid ester, and DL-limonene). The higher abundance of the sesquiterpenes in the HCT-LCT samples is caused by the loss of monoterpenes due to the stacked normalisation of the graph. Given that the loss in volatiles mainly concerns lighter compounds, the disproportion of the sesquiterpene fraction is less visible. In conclusion, it is possible to state that the whole extract generally maintained its original hops composition and peculiar fragrance without losing the major components responsible for the hop’s volatile fingerprint.

## 4. Discussion

This study has tested the ability of a MEFCUV system to recover the volatile compounds from two commercial hop varieties, and the results herein reported highlight the number of potential applications in flavouring areas. Since hydrodistillation is the common protocol for recovery/quantification of EO, this method was chosen to provide a comparative reference. The strength of this technology lies in its ability to extract volatiles from biomass under mild conditions and without organic solvents. This environmentally friendly approach is particularly useful for applications in the food industry, where the use of organic solvents is strictly regulated. In brewing applications, classic dry-hopping involves the soaking of the hop pellets at low temperatures (generally below 15 °C) for two to three weeks at the end of fermentation. This time-consuming process is inefficient and not cost-effective. Standard steam distillation for EO recovery is also time and energy consuming, plus the obtained volatiles bring several drawbacks in terms of dissolution. The lipophilic nature of EO poses two major problems for its application in beer products: low solubility and reduced foam stability. These two problems are closely related because, since EO are not water-soluble, their lower density causes them to migrate upward in aqueous media. When they reach the surface, the formation of a second lipophilic phase prevents foam stabilisation. These problems would not occur when LCT and HCT are used. This behaviour is due to the stable water suspension achieved during the evaporation and condensation cycles in the distillation columns. It should also be noted that even if the resulting aqueous components are close to the saturation limit, they cannot produce any lipophilic layers after adding them to the beer. This process offers the possibility of producing an extract that is extremely rich in sesquiterpene and monoterpene fractions since the depleted biomass has lost all of its flavour while at the same time achieving a reduction in the total volume ingested.

MEFCUV is highly versatile thanks to its industrial scalability and ability to produce extract fractions with different organoleptic properties. In a typical working day, a single operator can easily process 50–100 kg of hop pellets, and the extract collected can be easily stored and transported. MEFCUV is extremely versatile thanks to its industrial scalability and its ability to produce extract fractions with different organoleptic properties. In a typical working day, a single operator can easily process 50–100 kg of hop pellets, and the extract obtained can be easily stored and transported. Considering that the application requires storage at 4 °C, recovering the extract concentrate using MECUV can halve the volume of the refrigerator compared to pelletised hops, which also results in energy savings. The production yields achieved are sufficient to supply all types of craft breweries, and it should be noted that MEFCUV, with double volume and productivity, is already commercially available. In an industrial brewery, a more powerful chiller could be used, the volume of the feed tank could be increased, and even two extractors could be used in parallel. It is also important to emphasise the economic relevance of producing different extract fractions for original blends. The dispersion of terpenes in water also allows the extracts to be used in non-alcoholic beverages such as soft drinks, juices, integrators, and non-alcoholic beers. These aromatic terpene fractions can also be used in the cosmetic and perfume industries.

The HS-SPME/GC-MS technique was used for the identification and semi-quantification of volatiles in the collected hop samples. This analytical approach proved to be very efficient, with grey fibre application meeting expectations [43]. 

Work is in progress to confirm the enhanced shelf-life of the achieved aqueous extracts. Microbiological stability will be determined by storing samples at room temperature, 4 °C and −6 °C. In the meanwhile, it is possible to consider that samples stored at room temperature, after 4 months, maintained the same colour and clearness, do not presenting any mould or particle formations. A strong loss in the monoterpene fraction is expected at room temperature, while the refrigerated samples should fully preserve the compounds. Further studies are planned to introduce the different recovered fractions into a non-dry-hopped beer in order to evaluate differences with a traditional dry-hopped beer.

## Figures and Tables

**Table 1:** Average chemical composition of dried hop cones.

Constituent	Content (%)
Cellulose	45
Total resins	15–30
Proteins	15
Moisture	10
Ash	8
Polyphenols (tannins)	4
Essential oils (EO)	0.5–3
Monosaccharides	2
Pectins	2
Amino acids	0.1

**Table 2:** Results of the humidity level analysis performed on the pellet samples in triplicate.

Hops Variety	Humidity (*w*/*w*%)	S.D. (*w*/*w*%)
Citra	9.12	±0.13
Chinook	11.21	±0.32

**Table 3:** Temperature settings of each column for Chinook extraction.

	1st Column	2nd Column	3rd Column	4th Column
Top	25 °C	5 °C	−8 °C	−8 °C
Bottom	50 °C	25 °C	−8 °C	−8 °C
Pressure	−500 mbar	−500 mbar	−500 mbar	0 mbar

**Table 4:** Temperature settings of each column for Citra extraction.

	1st Column	2nd Column	3rd Column	4th Column
Top	30 °C	10 °C	−5 °C	−5 °C
Bottom	50 °C	30 °C	−5 °C	−5 °C
Pressure	−400 mbar	−400 mbar	−400 mbar	0 mbar

**Table 5:** Recovered condensed extract volume for each column at the end of the extraction process.

Hops	Fraction Volume (L)			
	C1	C2	C3	C4
Chinook	3.3	4.5	1.8	0.5
Citra	4.5	4.8	1.2	0.4

**Table 6:** Chinook compound distribution in the HCH and LCH samples showing triplicate average (AVG.) and standard deviation (S.D.).

Chinook Compounds	HCH	LCH
	AVG. ± S.D. (ppm)	AVG. ± S.D. (ppm)
β-Myrcene	14.1 ± 2.3	286.3 ± 44.9
Linalool-L	16.1 ±1.2	90.6 ± 4.8
Geraniol	2.9 ± 1.6	28.7 ± 2.1
α-Copaene	3.3 ±0.3	119.4 ± 23
*trans*-Caryophyllene	41.6 ± 1.5	992.4 ± 165.9
α-Humulene	87.4 ± 2.5	1955.4 ± 346.9
α-Amorphene	5.9 ± 0.3	350.5 ± 71
β-Selinene	6.6 ± 1.7	313.2 ± 64.8
α-Selinene	9.8 ± 1.5	388.5 ± 81.3
δ-Cadinene	7 ± 0.2	390.8 ± 81.7
Isoledene	2.1 ± 0.1	189.7 ± 41.5

**Table 7:** Citra compound distribution in HCH and LCH samples showing triplicate average (AVG.) and standard deviation (S.D.).

Citra Compounds	HCT	LCT
	AVG. ± S.D. (ppm)	AVG. ± S.D. (ppm)
β-Myrcene	54.6 ± 1.2	117.5 ± 11
Isobutyl isopentanoic acid ester	4.8 ± 0.7	12.5 ± 2
DL-Limonene	1.4 ± 0.1	8 ± 0.3
2-Nonanone	2.6 ± 0.3	6.2 ± 0.6
Linalool L	91.8 ± 12.5	267.5 ± 8
2-Decanone	2 ± 1	3 ± 0.4
Geraniol	6.4 ± 1.3	43.1 ± 3
2-Undecanone	25.1 ± 12	33.3 ± 1.6
2,6-Octadienoic acid, 3,7-dimethyl-, methyl ester	97.6 ± 17	100.5 ± 9.2
α-Copaene	9.5 ± 2.6	5.7 ± 1.1
*trans*-Caryophyllene	213.8 ± 36.9	155.2 ± 27.7
α-Humulene	392.6 ± 60.8	291.8 ± 40.4
α-Amorphene	25.5 ± 6	11.4 ± 2.4
β-Selinene	46.5 ± 4.5	32.9 ± 5.3
α-Selinene	51.7 ± 10.3	30.8 ± 5.5
α-Farnesene	12.1 ± 4.5	4.9 ± 0.7
Geranyl propionate	4.8 ± 1.2	0.7 ± 1.1
δ-Cadinene	26.4 ± 4.9	10.9 ± 2.2

## Data Availability

Data are contained in the article.

## Supplementary Materials

The following supporting information can be downloaded at: https://www.mdpi.com/article/10.3390/foods12081716/s1, Table S1: Schematic overview of the main compounds recovered from the extraction of hops’ EO [50,51]. Table S2: Data recovered from the HS-SPME/GC-MS of the three HCT samples analysed. Showing retention time (rt); percentage of quality recognition compared to the library (qual); total area of the peak (area) and the quantification of the compound; Table S3: Data recovered from the HS-SPME/GC-MS of the three LCT samples analysed. Showing retention time (rt); percentage of quality recognition compared to the library (qual); total area of the peak (area) and the quantification of the compound; Table S4: Data recovered from the HS-SPME/GC-MS of the three PCT samples analysed. Showing retention time (rt); percentage of quality recognition compared to the library (qual); total area of the peak (area) and the quantification of the compound; Table S5: Data recovered from the HS-SPME/GC-MS of the three PCT samples analysed. Showing retention time (rt); percentage of quality recognition compared to the library (qual); total area of the peak (area) and the quantification of the compound; Table S6: Data recovered from the HS-SPME/GC-MS of the three LCH samples analysed. Showing retention time (rt); percentage of quality recognition compared to the library (qual); total area of the peak (area) and the quantification of the compound; Table S7: Data recovered from the HS-SPME/GC-MS of the three PCH samples analysed. Showing retention time (rt); percentage of quality recognition compared to the library (qual); total area of the peak (area) and the quantification of the compound.
