# Supplementary material for: Industrial Multiple-Effect Fractional Condensation under Vacuum for the Recovery of Hop Terpene Fractions in Water
Source: Foods. 2023 Apr 20;12(8):1716. doi: 10.3390/foods12081716 (PMC10137393; doi:10.3390/foods12081716)
Supplement: Supplementary file 1 [file foods-12-01716-s001.zip › foods-2306198-supplementary.pdf]

Cathegory	Sub-cathegory	Compound
**Monoterpens**	Acyclic	β-Mircene, β-ocimene
	Monocyclic	D-Limonene
	Bicyclic	α-Pinene, β-pinene
	Monoterpene alcohols	R- and S-Linalol, Gerianol, β-citronellol, α-terpineol, myrcenol, nerol, myrtenol
	Monoterpene epoxides	*cis*-Linalool oxide
	Monoterpene ketons	(E)-β-damascenone, β-ionone
**Sesquiterpens**	Acyclic	β-Farnesene
	Monocyclic	α-Humulene
	Bicyclic	β-Caryophyllene, β-bergamotene, γ-muuroleene, β-eudesmene, δ-cadinene
	Sesq. alcohols	Humulol and humulenol II, nerolidol, δ-cadinol
	Monoepoxides	Humulene epoxide I, II, and III, caryophyllene oxide
**Ketones**		2-Nonanone, 2-undecanone, 2-dodecanone, 2-tridecanone
**Isobutyrates**		Octyl isobutyrate, geranyl isobutyrate, ethyl isobutyrate
**Esters**		Methyl octanoate, methyl 8-methylnonanoate, methyl (*E*)-4-decenoate, methyl geranate, geranyl propionate, methyl 3,6-dodecadienoate, ethyl 2-methylbutanoate, ethyl- 4-methylpentanoate, and ethyl 3-methylbutanoate

Table S1. Schematic overview of the main compounds recovered from the extraction of hops' EO [1], [2].

Volatile compounds HCH	vial 1					vial 2					vial 3					Mean	SD
	rt (min)	qual (%)	area	mg compound	ppm compound mg/kg aroma	rt (min)	qual (%)	area	mg compound	ppm compound mg/kg aroma	rt (min)	qual (%)	area	mg compound	ppm compound mg/kg aroma	ppm compound mg/kg aroma	
Toluene	***3.738***	***94***	***282022597***	***0.13005***		***3.607***	***94***	***295464665***	***0.1301***		***3.652***	***94***	***305732902***	***0.1301***			
β-Myrcene	10.602	96	17897703	0.00825	**16.51**	10.57	96	15768432.00	0.0069	**13.9**	10.606	96	14032455	0.0060	**11.9**	**14.1**	**2.3**
LINALOOL L	14.777	95	18746662	0.00864	**17.29**	14.768	96	18130064.00	0.0080	**16.0**	14.782	96	17609078	0.0075	**15.0**	**16.1**	**1.2**
Cyclopentasiloxane. decamethyl-											15.766	91	6758187	0.0029	**5.7**	**5.7**	**-**
GERANIOL	20.275	94	4367054	0.00201	**4.03**	20.284	93	2009120.00	0.0009	**1.8**						**2.9**	**1.6**
Methyl geranate						22.675	90	3268834.00	0.0014	**2.9**						**2.9**	**-**
α-Copaene	24.271	95	3787077	0.00175	3.49	24.271	96	3419945.00	0.0015	3.0	24.271	98	4102907	0.0017	3.5	**3.3**	**0.3**
trans-Caryophyllene	25.687	99	45622903	0.02104	**42.08**	25.687	99	45295221.00	0.0199	**39.9**	25.687	99	50227175	0.0214	**42.7**	**41.6**	**1.5**
α-Humulene	26.833	98	97658862	0.04503	**90.07**	26.838	98	96705046	0.0426	**85.1**	26.842	98	102214695	0.0435	**87.0**	**87.4**	**2.5**
α-Amorphene	27.440	98	6535544	0.00301	**6.03**	27.435	98	6241359.00	0.0027	**5.5**	27.435	99	7136906	0.0030	**6.1**	**5.9**	**0.3**
β-Selinene	27.889	99	5088165	0.00235	**4.69**	27.889	99	8604475	0.0038	**7.6**	27.889	99	8992058	0.0038	**7.6**	**6.6**	**1.7**
α-selinene	28.105	95	8760572	0.00404	**8.08**	28.101	99	11818810.00	0.0052	**10.4**	28.101	99	12811822	0.0054	**10.9**	**9.8**	**1.5**
γ. 2-cadinene	28.627	95	4941668	0.00228	**4.56**	28.622	96	4344489.00	0.0019	**3.8**	28.757	99	8940765	0.0038	**7.6**	**5.3**	**2.0**
δ-Cadinene	28.752	99	8329328	0.00384	**7.68**	28.757	99	8400058.00	0.0037	**7.4**						**7.5**	**0.2**
Isoledene	29.332	95	2427481	0.00112	**2.24**	29.341	95	2376833.00	0.0010	**2.1**	29.341	97	2334920	0.0010	**2.0**	**2.1**	**0.1**

Table S2. Data recovered from the HS-SPME/GC-MS of the three HCH samples analyzed. Showing retention time (rt); percentage of quality recognition compared to the library (qual); total area of the peak (area) and the quantification of the compound.

Volatile compounds LCH	vial 1					vial 2					vial 3					Mean	SD
	rt (min)	qual (%)	area	mg compound	ppm compound mg/kg aroma	rt (min)	qual (%)	area	mg compound	ppm compound mg/kg aroma	rt (min)	qual (%)	area	mg compound	ppm compound mg/kg aroma	ppm compound mg/kg aroma	
Toluene	***3.603***	***94***	***125851422***	***0.13005***		***3.661***	***94***	***178310129***	***0.1301***		***3.670***	***94***	***187582777***	***0.1301***	*260.1*		
Isoamyl propionate						10.017	90	5183946	0.0038	**7.6**	10.004	83	5258217	0.0036	**7.3**	**7.4**	**0.2**
β-Myrcene	10.566	96	117449970	0.12137	**242.74**	10.606	96	227909151	0.1662	**332.4**	10.592	96	204551040	0.1418	**283.6**	**286.3**	**44.9**
Propanoic acid. 2-methylbutyl ester	11.590	90	2532871	0.00262	**5.23**	11.496	90	4640416	0.0034	**6.8**	11.482	82	4732323	0.0033	**6.6**	**6.2**	**0.8**
ISOPENTYL ISOBUTANOATE	11.478	90	11485547	0.01187	**23.74**	11.609	90	20094034	0.0147	**29.3**	11.595	90	19987126	0.0139	**27.7**	**26.9**	**2.9**
DL-Limonene	12.067	80	7524197	0.00778	**15.55**	12.076	95	13347945	0.0097	**19.5**	12.067	94	12703647	0.0088	**17.6**	**17.5**	**2.0**
Methyl 6-methyl heptanoate	14.287	86	3432014	0.00355	**7.09**	14.292	80	5293105	0.0039	**7.7**	14.287	90	4961994	0.0034	**6.9**	**7.2**	**0.4**
2-Nonanone						14.503	94	3317589	0.0024	**4.8**	14.490	94	3640918	0.0025	**5.0**	**4.9**	**0.1**
LINALOOL L	14.764	97	45420282	0.04694	**93.87**	14.778	97	58384743	0.0426	**85.2**	14.773	97	67005073	0.0465	**92.9**	**90.6**	**4.8**
Pentyl 3-methylbutanoate	15.070	90	4937096	0.00510	**10.20**	15.074	91	7789528	0.0057	**11.4**	15.070	90	7566151	0.0052	**10.5**	**10.7**	**0.6**
GERANIOL	20.216	95	14883825	0.01538	**30.76**	20.217	95	19649490	0.0143	**28.7**	20.216	95	19223700	0.0133	**26.7**	**28.7**	**2.1**
2-Undecanone	21.655	97	21366099	0.02208	**44.16**	21.655	96	29260959	0.0213	**42.7**	21.659	96	21699763	0.0150	**30.1**	**39.0**	**7.7**
4-Decenoic acid. methyl ester	22.145	96	18806925	0.01943	**38.87**	22.149	97	27527248	0.0201	**40.2**	22.149	93	20500308	0.0142	**28.4**	**35.8**	**6.4**
2.6-Octadienoic acid. 3.7-dimethyl-. methyl ester	22.608	83	51060706	0.05276	**105.53**						22.612	83	52505918	0.0364	**72.8**	**89.2**	**23.1**
Ylangene	24.046	99	18064048	0.01867	**37.33**	24.051	99	27657704	0.0202	**40.3**	24.046	99	19513184	0.0135	**27.1**	**34.9**	**7.0**
α-Copaene	24.271	99	60917611	0.06295	**125.90**	24.285	99	94949485	0.0693	**138.5**	24.275	99	67656576	0.0469	**93.8**	**119.4**	**23.0**
10.10-Dimethyl-4-acetyl-tricyclo[5.2.1.0(1.5)]decane						25.211	81	4348477	0.0032	**6.3**						**6.3**	**-**
trans-Caryophyllene	25.781	99	511395410	0.52846	**1056.91**	25.826	99	765361199	0.5582	**1116.4**	25.795	99	579812125	0.4020	**804.0**	**992.4**	**165.9**
GERMACRENE-D/β cubebene	26.019	96	22390590	0.02314	**46.28**	26.042	98	36163181	0.0264	**52.8**	26.037	98	25249383	0.0175	**35.0**	**44.7**	**9.0**
α-Humulene	27.013	96	1040917012	1.07564	**2151.29**	27.076	96	1480849207	1.0801	**2160.1**	27.026	96	1121337457	0.7774	**1554.8**	**1955.4**	**346.9**
α-Amorphene	27.476	99	180650542	0.18668	**373.35**	27.503	99	279157207	0.2036	**407.2**	27.480	99	195299774	0.1354	**270.8**	**350.5**	**71.0**
α-Muurolene	27.588	98	20565565	0.02125	**42.50**	27.606	98	29758103	0.0217	**43.4**	27.593	97	20006582	0.0139	**27.7**	**37.9**	**8.8**
β-Selinene	27.925	99	161592346	0.16698	**333.97**	27.952	99	250306629	0.1826	**365.1**	27.930	99	173522769	0.1203	**240.6**	**313.2**	**64.8**
α-selinene	28.137	99	203872704	0.21067	**421.35**	28.164	98	307244066	0.2241	**448.2**	28.141	99	213435812	0.1480	**295.9**	**388.5**	**81.3**
α-Farnesene	28.375	98	9114635	0.00942	**18.84**	28.393	94	16759570	0.0122	**24.4**	28.379	91	10409172	0.0072	**14.4**	**19.2**	**5.0**
Geranyl propionate	28.501	90	10169803	0.01051	**21.02**	28.519	91	16617291	0.0121	**24.2**	28.510	87	9504737	0.0066	**13.2**	**19.5**	**5.7**
α-Amorphene	28.663	96	145057555	0.14990	**299.79**	28.685	97	220192619	0.1606	**321.2**	28.663	97	152201316	0.1055	**211.0**	**277.3**	**58.4**
δ-Cadinene	28.802	99	206141513	0.21302	**426.04**	28.838	99	307788919	0.2245	**449.0**	28.811	99	214530855	0.1487	**297.5**	**390.8**	**81.7**
1S.*cis*-CALAMENENE	28.923	97	67748892	0.07001	**140.02**	28.946	97	99361629	0.0725	**144.9**	28.932	97	68391834	0.0474	**94.8**	**126.6**	**27.6**
CADINA-1.4-DIENE	29.220	98	15014841	0.01552	**31.03**	29.238	98	25320702	0.0185	**36.9**	29.224	98	16639526	0.0115	**23.1**	**30.3**	**7.0**
Isoledene	29.355	97	99618757	0.10294	**205.88**	29.377	97	151252473	0.1103	**220.6**	29.364	97	102765033	0.0712	**142.5**	**189.7**	**41.5**
Selina-3.7(11)-diene	29.485	98	73879565	0.07634	**152.69**	29.508	98	110352665	0.0805	**161.0**	29.490	98	74363429	0.0516	**103.1**	**138.9**	**31.3**
1-Methyl-6-methylenebicyclo[3.2.0]heptane						29.800	91	5686293	0.0041	**8.3**						**8.3**	**-**
Caryophyllene oxide	30.699	95	7476032	0.00773	**15.45**	30.708	91	15253916	0.0111	**22.3**	30.699	95	12873199	0.0089	**17.8**	**18.5**	**3.4**
α-Caryophyllene	31.198	91	5387343	0.00557	**11.13**											**11.1**	**-**
caryophylla-4(12).8(13)-dien-5β-ol	32.299	93	3538508	0.00366	**7.31**											**7.3**	**-**
δ-gurjunene	32.820	81	13868928	0.01433	**28.66**											**28.7**	**-**
Ledene						32.825	90	16825783	0.0123	**24.5**						**24.5**	**-**
Eudesma-4(14).11-diene						33.252	94	10258788	0.0075	**15.0**						**15.0**	**-**

Table S3. Data recovered from the HS-SPME/GC-MS of the three LCH samples analyzed. Showing retention time (rt); percentage of quality recognition compared to the library (qual); total area of the peak (area) and the quantification of the compound.

Volatile compounds PCH	vial 1					vial 2					vial 3					Mean	SD
	rt (min)	qual (%)	area	mg SI	ppm compound mg/kg aroma	rt (min)	qual (%)	area	mg SI	ppm compound mg/kg aroma	rt (min)	qual (%)	area	mg SI	ppm compound mg/kg aroma	ppm compound mg/kg aroma	
**Toluene**	**3.585**	**94**	**211387515**	**0.2601**		**3.684**	**94**	**240470482**	**0.2601**		**3.616**	**94**	**251940178**	**0.2601**			
2-Pentanol. propanoate						10.035	80	7190810	0.1301	13005.0						-	-
β-Myrcene	10.574	96	175422431	0.2158	21584.7	10.619	96	191450630	0.2071	20707.9	10.584	96	155164565	0.1602	16019.0	19437.2	2992.5
Propanoic acid. 2-methyl-. 3-methylbutyl ester						*11.505*	*72*	4956337	0.0054	536.1					0.0	268.0	379.1
Propanoic acid. 2-methyl-. 2-methylbutyl este	11.590	90	14407173	0.0177	1772.7	11.622	90	18250771	0.0197	1974.1	*11.595*	*90*	*15769983*	0.0163	1628.1	1791.6	173.8
Methyl 6-methyl heptanoate	14.269	90	14091482	0.0173	1733.9	14.292	83	14866670	0.0161	1608.0	14.278	86	13089501	0.0135	1351.3	1564.4	195.0
LINALOOL L						14.795	83	4770891	0.0052	516.0						516.0	-
n-Amyl isovalerate	15.065	91	7885067	0.0097	970.2	15.074	90	9031027	0.0098	976.8	15.074	90	6701615	0.0069	691.9	879.6	162.6
Octanoic acid. methyl ester	15.708	92	7678262	0.0094	944.8	15.708	87	6867418	0.0074	742.8						843.8	142.8
Methyl 6-methyloctanoate	18.135	91	12534752	0.0154	1542.3	18.140	91	14592128	0.0158	1578.3	18.140	91	12539444	0.0129	1294.6	1471.7	154.5
Nonanoic acid. methyl ester (CAS)	19.272	94	4897887	0.0060	602.7	19.281	97	4653744	0.0050	503.4	19.286	14	3930536	0.0041	405.8	503.9	98.4
2-Undecanone	20.414	87	3947911	0.0049	485.8	20.414	87	4416523	0.0048	477.7	20.419	87	4862243	0.0050	502.0	488.5	12.4
E-Citral	20.904	97	3952548	0.0049	486.3	20.895	97	6938441	0.0075	750.5	20.904	96	5304460	0.0055	547.6	594.8	138.3
2-Undecanone (CAS)	21.659	96	12847934	0.0158	1580.9	21.659	97	14715212	0.0159	1591.6	21.664	97	13285530	0.0137	1371.6	1514.7	124.1
4-Decenoic acid. methyl ester	22.145	99	48537805	0.0597	5972.3	22.145	99	44884682	0.0485	4854.9	22.145	96	40717819	0.0420	4203.7	5010.3	894.5
2.6-Octadienoic acid. 3.7-dimethyl-. methyl ester	22.612	90	27935438	0.0344	3437.3	22.612	95	24952793	0.0270	2699.0	22.612	83	24375623	0.0252	2516.5	2884.3	487.6
α-Ylangene	24.051	98	8285233	0.0102	1019.4	24.051	96	6665445	0.0072	721.0	24.046	99	10880214	0.0112	1123.3	954.6	208.9
α-Copaene	24.266	99	24379715	0.0300	2999.8	24.266	99	19682346	0.0213	2128.9	24.266	98	33409013	0.0345	3449.1	2859.3	671.2
trans-Caryophyllene	25.714	99	180130593	0.2216	22164.0	25.709	99	149912543	0.1621	16215.0	25.709	99	177695232	0.1835	18345.0	18908.0	3014.2
GERMACRENE-D	26.019	98	12854865	0.0158	1581.7	26.019	98	16156425	0.0175	1747.5	26.019	98	18863708	0.0195	1947.5	1758.9	183.1
α-Humulene	26.900	98	401765887	0.4943	49434.9	26.878	97	271877127	0.2941	29407.0	26.883	98	297016263	0.3066	30663.6	36501.9	11218.0
α-Amorphene	27.444	99	56294628	0.0693	6926.7	27.444	99	55278032	0.0598	5979.0	27.449	99	76318131	0.0788	7879.0	6928.3	950.0
Naphthalene. 1.2.4a.5.6.8a-hexahydro-4.7-dimethyl-1-(1-methylethyl)-. (1α.4aα.8aα)-	27.557	98	5675128	0.0070	698.3	27.575	96	4816728	0.0052	521.0	27.566	96	7125162	0.0074	735.6	651.6	114.7
β-Selinene	27.889	99	29337219	0.0361	3609.8	27.894	99	29050396	0.0314	3142.2	27.894	99	40295008	0.0416	4160.0	3637.3	509.5
α-Cubebene	27.970	96	10214826	0.0126	1256.9	27.979	96	10320607	0.0112	1116.3	27.975	96	12106869	0.0125	1249.9	1207.7	79.2
α-selinene	28.101	99	58626027	0.0721	7213.6	28.101	99	31567365	0.0341	3414.4	28.105	98	68015246	0.0702	7021.8	5883.3	2140.2
α-Muurolene						28.163	99	19603627	0.0212	2120.4						2120.4	-
α-Fenchene	28.492	90	6546219	0.0081	805.5	28.487	92	6974692	0.0075	754.4						779.9	36.1
γ-Cadinene	28.627	97	53628510	0.0660	6598.7	28.626	97	55105469	0.0596	5960.4	28.631	97	73437057	0.0758	7581.6	6713.5	816.7
δ-Cadinene	28.766	99	63577998	0.0782	7822.9	28.757	99	20332093	0.0220	2199.2	28.761	99	21432650	0.0221	2212.7	4078.3	3243.0
1S.*cis*-CALAMENENE	28.896	97	16837791	0.0207	2071.8	28.896	97	12610036	0.0136	1363.9	28.896	97	16344637	0.0169	1687.4	1707.7	354.4
CADINA-1.4-DIENE	29.202	98	8526709	0.0105	1049.2	29.206	97	7731115	0.0084	836.2	29.202	91	10028507	0.0104	1035.3	973.6	119.2
α-cadinene	29.323	94	19517878	0.0240	2401.6	29.323	97	9824184	0.0106	1062.6	29.323	95	12562596	0.0130	1296.9	1587.0	715.1
(-)-Caryophyllene oxide						30.690	93	9647706	0.0104	1043.5	30.694	95	7515837	0.0078	775.9	909.7	189.2
E.E-α-FARNESENE						31.701	94	9140067	0.0099	988.6	31.697	86	6978020	0.0072	720.4	854.5	189.7
Alloaromadendrene	32.173	95	9071385	0.0112	1116.2				0.0000							1116.2	-
caryophylla-4(12).8(13)-dien-5β-ol						32.303	99	6756681	0.0073	730.8						730.8	-
δ-Cadinene	32.434	91	4161957	0.0051	512.1	32.434	94	6041919	0.0065	653.5	32.434	91	4854819	0.0050	501.2	555.6	85.0
α-Gurjunene	32.816	91	7248577	0.0089	891.9				0.0000							891.9	-
β-Panasinsene						32.820	91	11918780	0.0129	1289.2						1289.2	-

Table S4. Data recovered from the HS-SPME/GC-MS of the three PCH samples analyzed. Showing retention time (rt); percentage of quality recognition compared to the library (qual); total area of the peak (area) and the quantification of the compound.

Volatile compounds HCT	vial 1					vial 2					vial 3					Mean	SD
	rt (min)	qual (%)	area	Compound (mg)	ppm compound mg/kg aroma	rt (min)	qual (%)	area	Compound (mg)	ppm compound mg/kg aroma	rt (min)	qual (%)	area	Compound (mg)	ppm compound mg/kg aroma	ppm compound mg/kg aroma	
Toluene	***3.612***	***94***	***250634484***	***0.1300500000***		***3.567***	***94***	***200184832***	***0.1301***		***3.729***	***94***	***233364006***	***0.1301***			
β-Myrcene	10.584	96	53922239	0.0279793390	**55.9587**	10.548	96	41640996	0.0271	**54.1**	10.579	96	48285536	0.0269	**53.8**	***54.6***	***1.2***
ISOBUTYL ISOPENTANOIC ACID ESTER	11.617	90	4064899	0.0021092074	**4.2184**	11.600	90	4290252	0.0028	**5.6**	11.622	86	4214940	0.0023	**4.7**	***4.8***	***0.7***
DL-Limonene	12.054	95	1285021	0.0006667757	**1.3336**	12.054	95	1197021	0.0008	**1.6**	12.054	95	1297021	0.0007	**1.4**	***1.4***	***0.1***
2-Nonanone	14.508	97	2241566	0.0011631107	**2.3262**	14.503	91	2011816.00	0.0013	**2.6**	14.508	95	2573127.00	0.0014	**2.9**	***2.6***	***0.3***
LINALOOL L	14.782	97	80377767	0.0417066655	**83.4133**	14.764	97	66063155.00	0.0429	**85.8**	14.782	97	95322150.00	0.0531	**106.2**	***91.8***	***12.5***
Cyclopentasiloxane. decamethyl-	15.762	91	1612310	0.0008366004	**1.6732**	15.753	91	5700819.00	0.0037	**7.4**						***4.5***	***4.1***
2-Decanone	18.230	93	938692	0.0004870714	**0.9741**	18.234	93	1490420.00	0.0010	**1.9**	18.243	90	2675456	0.0015	**3.0**	***2.0***	***1.0***
GERANIOL	20.239	93	4798767	0.0024899991	**4.9800**	20.243	93	5650073	0.0037	**7.3**	20.243	93	6220200.00	0.0035	**6.9**	***6.4***	***1.3***
2-Undecanone	21.659	97	23514347	0.0122011975	**24.4024**	21.655	97	28818367	0.0187	**37.4**	21.668	97	12140436.00	0.0068	**13.5**	***25.1***	***12.0***
methyl Z-4-decenoate	22.149	99	16927205	0.0087832407	**17.5665**	22.149	95	17600666.00	0.0114	**22.9**	22.154	98	10241959	0.0057	**11.4**	***17.3***	***5.7***
2.6-Octadienoic acid. 3.7-dimethyl-. methyl ester						22.612	87	84381162.00	0.0548	**109.6**	22.608	95	76832895	0.0428	**85.6**	***97.6***	***17.0***
α-Ylangene	24.046	97	1833375	0.0009513073	**1.9026**						24.051	94	2518447.00	0.0014	**2.8**	***2.4***	***0.6***
α-Copaene	24.271	99	6470917	0.0033576495	**6.7153**	24.051	96	9096174.00	0.0059	**11.8**	24.266	99	8836037.00	0.0049	**9.8**	***9.5***	***2.6***
trans-Caryophyllene	25.709	99	165623069	0.0859390128	**171.8780**	25.709	99	175387205.00	0.1139	**227.9**	25.718	99	216787468.00	0.1208	**241.6**	***213.8***	***36.9***
α-Longipinene	26.662	81	1475857	0.0007657973	**1.5316**											***1.5***	***-***
α-Humulene	26.883	98	311522348	0.1616436841	**323.2874**	26.883	98	336173718.00	0.2184	**436.8**	26.891	98	374688994.00	0.2088	**417.6**	***392.6***	***60.8***
α-Amorphene	27.431	99	18614481	0.0096587398	**19.3175**	27.436	99	24120058.00	0.0157	**31.3**	27.435	99	23156908.00	0.0129	**25.8**	***25.5***	***6.0***
α-Muurolene	27.566	95	2341705	0.0012150712	**2.4301**	27.56	99	2708054.00	0.0018	**3.5**	27.561	97	2867046.00	0.0016	**3.2**	***3.0***	***0.6***
β-Selinene	27.894	99	41718353	0.0216469487	**43.2939**						27.894	99	44523756.00	0.0248	**49.6**	***46.5***	***4.5***
α-selinene	28.096	99	41067386	0.0213091729	**42.6183**	28.096	99	48342428	0.0314	**62.8**	28.101	99	44460331	0.0248	**49.6**	***51.7***	***10.3***
α-Farnesene	28.357	98	9885095	0.0051292088	**10.2584**	28.352	99	13249387	0.0086	**17.2**	28.366	94	7892694	0.0044	**8.8**	***12.1***	***4.5***
Geranyl propionate	28.505	90	3414128	0.0017715333	**3.5431**	28.496	87	4722141	0.0031	**6.1**						***4.8***	***1.8***
γ-Cadinene	28.618	96	12442515	0.0064562109	**12.9124**	28.618	97	14431622	0.0094	**18.8**	28.618	97	15826798	0.0088	**17.6**	***16.4***	***3.1***
δ-Cadinene	28.757	98	20518665	0.0106467887	**21.2936**	28.753	99	23876929	0.0155	**31.0**	28.752	99	24158535	0.0135	**26.9**	***26.4***	***4.9***
1S.CIS-CALAMENENE	28.896	95	5964677	0.0030949701	**6.1899**	28.896	96	6870007	0.0045	**8.9**	28.896	97	6314125	0.0035	**7.0**	***7.4***	***1.4***
ADINA-1.4-DIENE	29.211	95	1702299	0.0008832942	**1.7666**	29.207	98	2307782	0.0015	**3.0**	29.206	93	1875049	0.0010	**2.1**	***2.3***	***0.6***
α-cadinene	29.328	95	2358230	0.0012236457	**2.4473**	29.328	97	3234705	0.0021	**4.2**	29.328	97	2660744	0.0015	**3.0**	***3.2***	***0.9***
α-CALACORENE	31.521	83	2778210	0.0014415662	**2.8831**	29.512	97	1467611	0.0010	**1.9**						***2.4***	***0.7***
Trans-γ-BISABOLENE						31.198	92	3337075	0.0022	**4.3**						***4.3***	***-***
3-Octyne. 5-methyl-											31.821	86	4068303	0.0023	**4.5**	***4.5***	***-***
7-Hexadecyn-1-ol						33.144	83	1866322	0.0012	**2.4**						***2.4***	***-***
Spiro[5.6]dodecane						33.324	90	1496771	0.0010	**1.9**						***1.9***	***-***

Table S5. Data recovered from the HS-SPME/GC-MS of the three HCT samples analyzed. Showing retention time (rt); percentage of quality recognition compared to the library (qual); total area of the peak (area) and the quantification of the compound.

Volatile compounds LCT	vial 1					vial 2					vial 3					Mean	SD
	rt (min)	qual (%)	area	Compound (mg)`	ppm compound mg/kg aroma	rt (min)	qual (%)	area	Compound (mg)`	ppm compound mg/kg aroma	rt (min)	qual (%)	area	Compound (mg)	ppm compound mg/kg aroma	ppm compound mg/kg aroma	
Toluene	***3.553***	***94***	***191619736***	***0.1301***		***3.724***	***94***	***222157755***	***0.1301***		***3.585***	***94***	***204372693***	***0.1301***			
β-Myrcene	10.543	96	87659274	0.0595	**119.0**	10.579	96	90316911	0.0529		10.552	96	100309412	0.0638	**127.7**	**117.5**	**11.0**
ISOBUTYL ISOPENTANOIC ACID ESTER	11.581	90	10055595	0.0068	**13.6**	11.608	90	8705249	0.0051	**10.2**	11.585	90	10798443	0.0069	**13.7**	**12.5**	**2.0**
dl-Limonene	12.031	98	6006725	0.0041	**8.2**						12.04	97	6100988	0.0039	**7.8**	**8.0**	**0.3**
β-Phellandrene						12.125	96	4989908	0.0029	**5.8**						**5.8**	**-**
β-OCIMENE Y	12.741	96	3207075	0.0022	**4.4**						12.746	90	3330254	0.0021	**4.2**	**4.3**	**0.1**
2-Nonanone	14.481	97	4824210	0.0033	**6.5**	14.494	97	4722402	0.0028	**5.5**	14.485	97	5234858	0.0033	**6.7**	**6.2**	**0.6**
LINALOOL L	14.809	97	191123900	0.1297	**259.4**	14.831	97	228545346	0.1338	**267.6**	14.822	97	216488063	0.1378	**275.5**	**267.5**	**8.0**
BORNEOL L	17.425	90	3164748	0.0021	**4.3**	17.438	95	3625779	0.0021	**4.2**	17.43	90	3569743	0.0023	**4.5**	**4.4**	**0.2**
4-Terpineol						17.740	99	2527846	0.0015	**3.0**	17.74	97	2447329	0.0016	**3.1**	**3.0**	**0.1**
2-Decanone	18.194	93	2407275	0.0016	**3.3**	18.198	94	2215267	0.0013	**2.6**	18.189	97	2488409	0.0016	**3.2**	**3.0**	**0.4**
α-TERPINEOL	18.306	91	4685742	0.0032	**6.4**	18.310	91	5293229	0.0031	**6.2**	18.306	91	2488409	0.0016	**3.2**	**5.2**	**1.8**
Nerol	19.313	93	4943728	0.0034	**6.7**	19.317	96	5290019	0.0031	**6.2**	19.317	93	6024749	0.0038	**7.7**	**6.9**	**0.7**
GERANIOL	20.212	97	29374908	0.0199	**39.9**	20.216	95	37432781	0.0219	**43.8**	20.212	95	35874990	0.0228	**45.7**	**43.1**	**3.0**
2-Undecanone	21.097	97	25476226	0.0173	**34.6**	21.659	97	26944310	0.0158	**31.5**	21.655	97	26435490	0.0168	**33.6**	**33.3**	**1.6**
4-Decenoic acid. methyl ester	22.154	96	9203429	0.0062	**12.5**	22.158	97	8959084	0.0052	**10.5**	22.154	95	9244714	0.0059	**11.8**	**11.6**	**1.0**
2.6-Octadienoic acid. 3.7-dimethyl-. methyl ester	22.612	83	78632644	0.0534	**106.7**	22.617	95	76874870	0.0450	**90.0**	22.612	83	82360158	0.0524	**104.8**	**100.5**	**9.2**
α-Copaene	24.271	95	3406438	0.0023	**4.6**	24.266	99	4796853	0.0028	**5.6**	24.266	99	5284990	0.0034	**6.7**	**5.7**	**1.1**
trans-Caryophyllene	25.696	99	94979873	0.0645	**128.9**	25.705	99	130297434	0.0763	**152.6**	25.705	99	144641145	0.0920	**184.1**	**155.2**	**27.7**
α-Humulene	26.851	98	188877535	0.1282	**256.4**	26.873	99	241819629	0.1416	**283.1**	26.869	98	263873270	0.1679	**335.8**	**291.8**	**40.4**
α-Amorphene	27.435	99	6629811	0.0045	**9.0**	27.435	98	9800971	0.0057	**11.5**	27.431	99	10775104	0.0069	**13.7**	**11.4**	**2.4**
β-Selinene	27.885	99	20668421	0.0140	**28.1**	27.889	99	27414686	0.0160	**32.1**	27.894	99	30347689	0.0193	**38.6**	**32.9**	**5.3**
α-selinene	28.096	99	18954034	0.0129	**25.7**	28.101	99	25653497	0.0150	**30.0**	28.096	99	28829130	0.0183	**36.7**	**30.8**	**5.5**
α.-Farnesene	28.366	96	3301202	0.0022	**4.5**	28.370	98	3872023	0.0023	**4.5**	28.361	99	4507707	0.0029	**5.7**	**4.9**	**0.7**
Geranyl propionate						28.505	87	1695368	0.0010	**2.0**						**2.0**	**-**
δ-Cadinene	28.752	99	7264272	0.0049	**9.9**	28.752	99	10194378	0.0060	**11.9**	28.757	99	11190226	0.0071	**14.2**	**10.9**	**2.2**

Table S6. Data recovered from the HS-SPME/GC-MS of the three LCT samples analyzed. Showing retention time (rt); percentage of quality recognition compared to the library (qual); total area of the peak (area) and the quantification of the compound.

Volatile compounds PCT	vial 1					vial 2					vial 3					Mean	SD
	rt (min)	qual (%)	area	Compound (mg)`	ppm compound mg/kg aroma	rt (min)	qual (%)	area	Compound (mg)`	ppm compound mg/kg aroma	rt (min)	qual (%)	area	Compound (mg)`	ppm compound mg/kg aroma	ppm compound mg/kg aroma	
Toluene	***3.715***	***94***	***270817180***	***0.2601***		***3.450***	***94***	***359153466***	***0.2601***		***3.706***	***94***	***343024554***	***0.2601***			
β-Myrcene	10.642	96	474950596	0.4562	**45615.5**	10.570	96	310062497	0.2245	**22454.8**	10.629	96	329971523	0.2502	**25020.2**	**31030.2**	**12696.2**
Heptane. 4-methyl-						11.465	72	3467450	0.0025	**251.1**						**251.1**	
ISOBUTYL ISOPENTANOIC ACID ESTER	11.595	90	15662653	0.0150	**1504.3**	11.568	90	15208655	0.0110	**1101.4**	11.604	90	14639539	0.0111	**1110.1**	**1238.6**	**230.1**
DL-Limonene	12.040	99	13299262	0.0128	**1277.3**	12.018	99	9118286	0.0066	**660.3**	12.053	99	8486572	0.0064	**643.5**	**860.4**	**361.2**
LINALOOL L	14.773	97	13888987	0.0133	**1333.9**	14.760	96	16339205	0.0118	**1183.3**	14.773	97	18721555	0.0142	**1419.6**	**1312.3**	**119.6**
Octanoic acid. methyl ester	15.690	94	6329451	0.0061	**607.9**	15.695	94	7286480	0.0053	**527.7**						**567.8**	**56.7**
2-Undecanone	21.655	96	32996212	0.0317	**3169.0**	21.655	97	39287302	0.0285	**2845.2**	21.655	97	42695496	0.0324	**3237.4**	**3083.9**	**209.5**
4-Decenoic acid. methyl ester	22.140	91	32183926	0.0309	**3091.0**	22.140	91	35341113	0.0256	**2559.4**	22.145	95	38834393	0.0294	**2944.6**	**2865.0**	**274.6**
2.6-Octadienoic acid. 3.7-dimethyl-. methyl ester	22.612	83	78854375	0.0757	**7573.4**	22.612	95	86971363	0.0630	**6298.5**	22.617	83	95822803	0.0727	**7265.8**	**7045.9**	**665.3**
α-Copaene	24.266	96	6309392	0.0061	**606.0**	24.267	96	4655690	0.0034	**337.2**	24.271	98	5041446	0.0038	**382.3**	**441.8**	**144.0**
trans-Caryophyllene	25.705	99	140930851	0.1354	**13535.4**	25.700	99	115058707	0.0833	**8332.6**	25.700	99	127998977	0.0971	**9705.6**	**10524.5**	**2696.3**
α-Humulene	26.869	97	245470593	0.2358	**23575.6**	26.860	98	216574256	0.1568	**15684.4**	26.869	98	239531102	0.1816	**18162.6**	**19140.9**	**4035.6**
α-Amorphene	27.431	99	10269331	0.0099	**986.3**	27.431	98	8437597	0.0061	**611.1**	27.435	98	10398835	0.0079	**788.5**	**795.3**	**187.7**
β-Selinene	27.894	99	34111900	0.0328	**3276.2**	27.890	99	27713202	0.0201	**2007.0**	27.894	99	31919142	0.0242	**2420.3**	**2567.8**	**647.3**
α-selinene	28.092	99	33905998	0.0326	**3256.4**	28.096	96	29833523	0.0216	**2160.6**	28.101	98	32903390	0.0249	**2494.9**	**2637.3**	**561.6**
α-Farnesene	28.352	99	7605074	0.0073	**730.4**	28.353	96	5035740	0.0036	**364.7**	28.352	96	7758084	0.0059	**588.3**	**561.1**	**184.4**
Geranyl propionate						28.492	87	3888620	0.0028	**281.6**	28.487	87	5215550	0.0040	**395.5**	**338.5**	**80.5**
γ-Cadinene	28.613	97	8233511	0.0079	**790.8**	28.622	97	6844792	0.0050	**495.7**	28.618	98	7430283	0.0056	**563.4**	**616.6**	**154.6**
δ-Cadinene	28.748	99	12118292	0.0116	**1163.9**	28.753	99	9035446	0.0065	**654.3**	28.753	99	11625278	0.0088	**881.5**	**899.9**	**255.3**

Table S7. Data recovered from the HS-SPME/GC-MS of the three PCT samples analyzed. Showing retention time (rt); percentage of quality recognition compared to the library (qual); total area of the peak (area) and the quantification of the compound.

References

[1] K. Rutnik, M. Knez Hrnčič, and I. Jože Košir, "Hop Essential Oil: Chemical Composition, Extraction, Analysis, and Applications," *Food Rev. Int.*, vol. 38, no. sup1, pp. 529–551, Nov. 2022, doi: 10.1080/87559129.2021.1874413.

[2] C. Dietz, D. Cook, C. Wilson, P. Oliveira, and R. Ford, "Exploring the multisensory perception of terpene alcohol and sesquiterpene rich hop extracts in lager style beer," *Food Res. Int.*, vol. 148, p. 110598, Oct. 2021, doi: 10.1016/j.foodres.2021.110598.
